# Sorption of Hydrophobic Organic Compounds on Natural Sorbents and Organoclays from Aqueous and Non-Aqueous Solutions: A Mini-Review

**DOI:** 10.3390/ijerph110505020

**Published:** 2014-05-09

**Authors:** Francis Moyo, Roman Tandlich, Brendan S. Wilhelmi, Stefan Balaz

**Affiliations:** 1 Division of Pharmaceutical Chemistry, Faculty of Pharmacy, Rhodes University, P.O. Box 94, Grahamstown 6140, South Africa; E-Mail: fmoyo30@yahoo.com; 2 Department of Biochemistry, Microbiology and Biotechnology, Rhodes University, P.O. Box 94, Grahamstown 6140, South Africa; E-Mail: b.wilhelmi@ru.ac.za; 3 Department of Pharmaceutical Sciences, Albany College of Pharmacy and Health Sciences Vermont Campus, 261 Mountain View Drive, Colchester, VT 05446, USA; E-Mail: stefan.balaz@acphs.edu

**Keywords:** kaolinite, siloxane, X-ray, sorption isotherms, Collander equation, hydrophobic organic compounds, non-aqueous phase liquids

## Abstract

Renewed focus on the sorption of hydrophobic organic chemicals (HOCs) onto mineral surfaces and soil components is required due to the increased and wider range of organic pollutants being released into the environment. This mini-review examines the possibility of the contribution and mechanism of HOC sorption onto clay mineral sorbents such as kaolinite, and soil organic matter and the possible role of both in the prevention of environmental contamination by HOCs. Literature data indicates that certain siloxane surfaces can be hydrophobic. Therefore soils can retain HOCs even at low soil organic levels and the extent will depend on the structure of the pollutant and the type and concentration of clay minerals in the sorbent. Clay minerals are wettable by nonpolar solvents and so sorption of HOCs onto them from aqueous and non-aqueous solutions is possible. This is important for two reasons: firstly, the movement and remediation of soil environments will be a function of the concentration and type of clay minerals in the soil. Secondly, low-cost sorbents such as kaolinite and expandable clays can be added to soils or contaminated environments as temporary retention barriers for HOCs. Inorganic cations sorbed onto the kaolinite have a strong influence on the rate and extent of sorption of hydrophobic organic pollutants onto kaolinite. Structural sorbate classes that can be retained by the kaolinite matrix are limited by hydrogen bonding between hydroxyl groups of the octahedral alumosilicate sheet and the tetrahedral sheet with silicon. Soil organic carbon plays a key role in the sorption of HOCs onto soils, but the extent will be strongly affected by the structure of the organic soil matter and the presence of soot. Structural characterisation of soil organic matter in a particular soil should be conducted during a particular contamination event. Contamination by mining extractants and antibiotics will require renewed focus on the use of the QSAR approaches in the context of the sorption of HOCs onto clay minerals from aqueous and non-aqueous solutions.

## 1. Introduction

Increased industrial use of hydrophobic organic chemicals (HOCs), along with increased exposure of environmental and agricultural systems to them, has occurred in recent years [1,2]. HOCs are often found in topsoil [3,4] and aquatic systems where they end up after accidental spillages or leaks from waste disposal sites [5,6,7]. Examples of the spillages can arise from the following considerations: in North America alone, 1,000 different mixtures of chemicals, including non-aqueous phase liquids (NAPLs) are transported on regular basis using rail tankers [8]. When there are spillages from the HOC-containing rail tankers, e.g., after an accident, the soil and the underground water are impacted [9], leading to costly clean-up operations [10]. Such spillages can result in groundwater contamination [7,11,12]. HOCs generally have low aqueous solubilities [13]. This, in combination with varying NAPL/HOC densities in comparison to water [14,15] leads to the formation of light or dense non-aqueous phase liquids (LNAPL/LNALs or DNAPL/DNAPLs) after a contamination event [16].

Examples of LNALPs include gasoline, while DNALPs are generally composed of chemicals such as trichloroethylene [8,17]. The LNAPLs or DNAPLs get trapped in the soil matrices as they migrate into environmental compartments such as the soil subsurface. As a result, LNAPLs end up floating on top of the water table, while DNAPLs sink to the bottom of groundwater reservoirs [18]. HOCs contained in the LNAPLs or DNAPLs partition into the groundwater and this leads to long-term pollution of groundwater and the vadose zone due to capillary rise of the HOCs [8,11,12]. If the soil hydraulic conductivity changes with soil depth, the DNAPLs saturate relatively permeable soil layers and flow laterally along the low-permeability ones made of clay minerals [19]. This results in the formation of continuous LNAPLs or DNAPLs in contaminated soils [20]. DNAPLs often contain trace contaminants such as polyaromatic hydrocarbons [18]. A combination of these two factors can lead DNAPLs to form a continuous source of soil and groundwater contamination. This also alters the HOCs’ macropore transport in soil environments due to HOCs’ dissolution in LNAPLs or DNAPLs; and the resulting shifts in HOC sorption patterns [20].

If the retention of DNAPLs in soils increases, the probability of human exposure to HOCs is heightened [21], which is undesirable from a public health point of view. Persistence of NAPLs is a worldwide problem, but in developing countries there has been little attention to managing it [16]. At HOC soil concentrations of 50–100 g/kg soil dry weight, the HOCs’ soil mobility becomes independent of the soil composition as demonstrated for BTEX compounds [14]. Thus, tracing HOC and LNAPL/DNAPL movement through soils, e.g., using the partitioning tracer test [22], is critical for understanding the role of soil organic and mineral components in the sorption of HOCs in soils. Clay minerals form a significant part of the structure of soil particles. This, in combination with the above-mentioned facts, indicates the importance of understanding of the role of clay minerals in the fate of HOCs in soil environments. Examples of clay minerals include kaolinite and montmorillonite which are commercially available and cheap from the remediation point of the view. Sorption onto clay sorbents could thus be applied in the removal of HOCs from contaminated environmental compartments, particularly in developing countries where mining constitutes a major part of the economy [23]; and where the costs of remedial operations are often prohibitive. The HOCs’ soil sorption is a function of the sorbent properties such as mineral content and the soil organic carbon concentrations. Covalent structure and the physico-chemical properties of HOCs are also important [24,25]. The apparent sorption equilibrium is reached once the HOC concentration in the two phases becomes independent of the sorbate-sorbent contact time [21,26,27]. The ratio of the mineral and soil organic matter as well as the contact time will have a strong influence on the HOC sorption in soil environments [26]. Various sorption mechanisms and structural features of soil particulate matter are important in this context.

Pu and Cutright [28] studied the sorption of pentachlorophenol onto a silty loam with a clay mineral weight fraction of 10% and a sandy-clay-loam with a clay content of 18% (w/w). Two artificial soils were mixed in the laboratory to obtain a more fundamental understanding of the sorption process for pentachlorophenol [28]. The silt loam had a higher sorption affinity for pentachlorophenol than the sandy loam that had a high content of kaolinite [28]. Sorption hysteresis was observed with sorption of pentachlorophenol onto all studied matrices. The sandy loam retained 74 to 86% of the sorbed amount of pentachlorophenol, while the silty loam with low kaolinite content retained 84 to 96% of the sorbed amount of pentachlorophenol [28]. These results seem to suggest that the extent of pentachlorophenol sorption onto soils is a function of the presence and type of clay mineral(s) in the particular soil. At the same time, the silt loam contained less kaolinite and had higher sorption affinity for pentachlorophenol than sandy-clay loam. Therefore the experimental results from this study seem to indicate that the majority of the sorption in clay rich soils is likely taking place on the internal crystal lattice surface, *i.e.*, on expandable clay minerals [28]. This conclusion was supported by the results from the artificial (in-the-laboratory mixed synthetic) soils that indicated the critical role of expandable clay minerals in pentachlorophenol sorption onto the studied soils. Pentachlorophenol is a highly hydrophobic HOC, but it also contains a phenolic group which is acidic in nature. This, in combination with the above-mentioned sorption data, suggests that the sorption of HOC onto soils and natural sorbents will depend on the soil properties, as well as the HOC’s structure. The nature of the clay minerals and the organic matter present in the particular soil will then dictate the sorption extent and mechanism in the given soil/natural sorbent. In the case of the pentachlorophenol, such a mechanism can include a combination of ion exchange and partitioning. Each combination of HOC and sorbent will be different in this regard. 

The sorption affinity of clay minerals towards HOCs can be modified using a suspension of the solid matrix in water and the subsequent exposure of the clay mineral surface to the solution of a surfactant that adsorbs onto the clay particle surface [29,30]. The rate of ion exchange of the inorganic cation for its organic counterpart will depend on the valence and atomic radius of the exchanged inorganic cation [31]. Cations of alkaline metals such as Na^+ ^are exchanged more readily, while the divalent cations such as the alkaline earth metal Ca^2+^ require full saturation of the clay surface with the organic cation to readily achieve ion exchange [31]. The efficiency of the cation exchange and achievement of the stoichiometric ion exchange depends on the solvent in which the organic cations is dissolved and that is used to wet the clay mineral surface [31,32]. The efficiency of this process increases with increasing water-miscibility of the wetting solvent [31,32].

In the sorption of quarternary alkyl ammonium salts onto expandable clay minerals, the majority of the specific (sorption) surface area was found to occur on the internal surfaces, *i.e.*, between the crystal lattice layers [26]. As a result, the majority of the surfactant sorption is going to take place on these internal surfaces [33]. Therefore the more surfactant, *i.e.*, the more quarternary alkyl ammonium salts are sorbed onto the montmorillonite matrix, the more the basal spacing of its crystal lattice will increase [31,34]. This section indicates that a vast amount of information on the sorption of HOCs onto natural sorbents is available in scientific literature [35,36,37,38,39,40,41]. The current article presents a mini-review of this knowledge about the sorption of HOCs onto soils and clay minerals. Specific focus will be placed on the HOCs’ sorption onto clay minerals from aqueous and non-aqueous solutions, with links made to the role of soil organic carbon. Some information on the knowledge gaps is presented and further research directions proposed. The article is part of an on-going project into the use of low-cost sorbents for the treatment of mining waste side-streams and similar types of wastewaters. 

## 2. Sorption Isotherms and Sorption Coefficient

The standard characterisation of an HOC’s sorption behaviour is *via* the corresponding sorption isotherm. The following mathematical models have been derived and used to describe the HOC sorption onto soils [42,43,44,45]: the Langmuir isotherm, the Brunauer–Emmett–Teller isotherm (BET), the Gibbs isotherm, the Freundlich isotherm and the linear isotherm. Generally speaking, all these models relate the dissolved sorbate concentration *C*_e_ (unit μg/mL or mg/L) and the sorbed concentration of the sorbate *q*_e_ (unit mg/kg dry weight of soil sorbent or μg/mg dry weight of soil sorbent; and the molar version has the unit of mmol/g or mmol/kg). Different models provide insight into the sorption mechanism of the given HOC onto a particular soil/clay mineral [26]. This in turn allows making predictions about the environmental concentrations of HOCs and the implication for environmental management. The following sections provide an outline of the most common models that have been applied to sorption of the HOCs in soils. 

### 2.1. The Langmuir Isotherm

The Langmuir isotherm was first used to describe gaseous sorption onto solids. It has been adopted to describe the sorption of chemical substances by natural solids. It describes the sorption of a sorbate onto the surface site of the sorbent takes place at specific homogenous sorption sites within the sorbent [46,47]. The theory is based on the assumption that once a solute molecule occupies a site, no further adsorption can take place at that site [46], *i.e.*, so-called the monolayer adsorption [48]. It also assumes that the surface of the adsorbent is in contact with a solution containing a sorbate molecule which is strongly attracted to its surface. It is suitable to for example to describe the HOC sorption onto humic substances. These cross-linked humic structures lead to minimum flexibility of the humic structure to accommodate the sorbent molecules with the increase in the solute concentration [27]. The monolayer adsorption chemical reaction describing the adsorption of sorbate to sorbent can be represented by the Equation (1) [38] below:

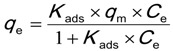
(1)
where *K_ads_* is the equilibrium adsorption coefficient (mL/g), *q*_m_ is the maximum values of *q*_e _(unit of μg/g dry weight of sorbent of mg/kg dry weight of sorbent) and *C*_e _has the same meaning of the stated above (mmol/L or mg/L). The other assumptions for the Langmuir isotherm are that there are no sorbate-sorbate interactions and that once adsorbed, the molecule becomes immobile.

The antibiotic enrofloxacin was shown to adhere to the Langmuir sorption isotherm with sorption capacities of 667, 228 and 20 mmol/kg clay mineral for SWy-2 montmorillonite, IMt-2 illite and KGa-1b kaolinite, respectively [49]. The sorption kinetics followed the pseudo-second order and the fastest uptake was observed on the KGa-1b kaolinite with a respective mass-based rate constant of 0.73 kg/mmol/h [49]. The enrofloxacin sorption occurred mainly via cation exchange on the SWy-2 montmorillonite and the IMt-2 illite [49]. For all three, the non-electrostatic interactions took over as the dominant interactions in the enrofloxacin sorption if the pH of the aqueous phase exceeded 7.0 [49]. The enrofloxacin intercalation, *i.e.*, sorption onto the internal surfaces of the SWy-2 montmorillonite was confirmed using XRD analyses [49].

Enrofloxacin is a second-generation fluroquinolone antibiotic [50]. It has been shown to be an HOC as the logarithm of its partition coefficient in phospholipid/water systems has been shown to range from 3.94 to 5.18 [50]. At the same time, the compound has two ionisable groups and these are a carboxylic acid group with a pKa value of 6.17 ± 0.01 and a piperazinyl group with a respective pKa = 7.72 ± 0.01 [51]. Thus the above-mentioned results and the physical constants for enrofloxacin indicate that the sorption of this antibiotic onto clay minerals might be a combination of interaction of the ionised groups, *i.e.*, the carboxylic and piperazinyl, with the charged clay surface and the hydrophobic spaces inside the crystal lattice of the clay minerals. Hydrophobic surfaces have been indicated to the located on the atoms of O which are in turn located inside the interlayer spaces in the clay crystal lattices [52]. 

In a mixed dye solution with methylene blue and malachite green, competitive sorption was observed with the sorption isotherms the Langmuir model and the competitive Langmuir model [53]. The main sorption mechanism to both the kaolinite matrix and tuff was ion exchange which accounted for 61%–79% of sorption uptake [53]. The *q*_m_ *o*f a mixture of clay minerals with 80% of kaolinite (w/w) has been reported to be equal to 0.77 mmol/g for methylene blue and 0.64 mmol/g for malachite green [53]. In the same study, the zeolite mineral tuff which is composed of 95% of philipsite reached *q*_m_ values of 0.66 mmol/g of methylene blue and 1.22 mmol/g for malachite green [53]. The experiments were conducted with a mean sorbent particle size equal to less than 45 μm and the temperature of the experiments was 20.0 °C [53]. The initial sorbate concentrations were set at 1.0 g/L and the pH of the dye solutions was equal to 7.0 [53].

Zhang *et al*. [34] studied the pH dependence of tylosin sorption onto kaolinite and montmorillonite. The sorption molecular mechanism was a combination of ion exchange and hydrophobic interactions. The observations from the studies on enrofloxacin, methylene blue, malachite green and tylosin sorption indicate that a wide range of organic compounds is likely to experience the dual-mode sorption model where the sorption is the results of several sorption mechanisms. The first one was the ion-exchange component that adhered to the “site-limiting Langmuir isotherm” [34]. The second component was a linear isotherm describing hydrophobic interactions [34]. This type of isotherm is summarised in Equation (2):

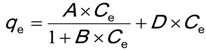
(2)

In Equation (2), *A*, *B* and *D* are adjustable parameters with units analogous to those of the adjustable parameters in Equation (1) (*A* for *K*_ads_ × *q*_m_ and *B* for *K*_ads_). *D* has the same unit as the sorption partition coefficient in the linear sorption isotherm (see Equation (4) for details). The X-ray diffraction results showed that tylosin molecules were intercalated inside the montmorillonite crystal lattice [34]. Hydrophobicity of the clay mineral surfaces changed as a function of the crystal lattice and interactions between the layers inside it, *i.e.*, the degree of the sorbate uptake by the sorbent [26].

A similar mechanism of sorption as described in Equation (2) could be used to explain the data of Figueroa *et al*. [54] who studied the sorption of oxytetracycline, chlorotetracycline and tetracycline onto kaolinite and montmorillonite. Sorption occurred via cation exchange and the surface complexation of antibiotic zwitterions [54]. The complexation of zwitterions occurred in an agonistic fashion with sorption of H^+^, with the extent of sorption being higher on acidic clays and independent of the ionic strength of the dissolved phase [54]. If the pH was above 7.0, then oxytetracycline sorption was stimulated by the presence of Ca^2+^, most likely due to a “surface-bridging mechanism” [54]. Such data could be explained by Equation (2) and the linear part could be accounted for by antibiotic precipitation with Ca^2+^. This in turn provides a possible mechanism for removal of antibiotics from the aqueous phase as such precipitation has been well documented in the pharmaceutical literature [55,56,57,58]. If the cation exchange capacity normalisation was performed and the ionic strength was low, then montmorillonite had a higher sorption affinity for the studied antibiotics than kaolinite, independently of the aqueous phase pH (see Section 5 for details on pH effects) [54].

Tylosin and antibiotics are antimicrobial agents which are often used in the dairy industry [59] and in agriculture as growth promoters [60]. Significant amounts of antibiotics are excreted without significant covalent modifications in the urine and faeces of human patients who are on antibiotic drug regimens [55,56,57,58], therefore finite amounts are discharged in wastewaters and reach municipal [61] and hospital sewage [62] treatment systems. Inside wastewater treatment systems, antimicrobial agent molecules can be retained on the particles of activated sludge or they pass through the systems unchanged and can become sorbed in downstream environmental compartments. Their sorption characteristics are likely to vary significantly due to their wide range of structural features and sorption-relevant properties.

A relevant example can be made using the comparison of the properties of ampicillin (CAS Number: 69-53-4) and sulfamethoxazole (CAS Number: 723-46-6). Their hydrophobicity was estimated by the authors through calculations of the logarithm of the 1-octanol/water partition coefficient (SRC LOGKOW software package, version 1.54) which equals 0.58 for sulfamethoxazole and 1.45 for ampicillin. At the same time, the aqueous solubility was estimated using the SRC WSKOW version 1.56 software package and found to be equal to 439.3 mg/L for ampicillin and 3,025 mg/L for sulfamethoxazole. These characteristics, together with the ionisability of the HOC molecule(s) and the sorbent surface or structure, will determine the interaction with the hydrophobic parts of the kaolinite crystal lattice [26] and the overall extent of sorption [21]. Given these differences, the quantitative-structure activity relationships (QSARs) should be used to predict sorption behaviour and parameters of HOCs on kaolinite (clay minerals). This approach will be outlined in Section 5.3 of this review (see below). 

### 2.2. The Freundlich Isotherm

Experimental HOC sorption data can also be fitted by the Freundlich isotherm as shown in Equation (3) below:

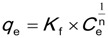
(3)
where *q*_e _and *C*_e _have the same units and meaning as in the case of the Langmuir isotherm as described in Section 2.1. At the same time, *K_f_* is the Freundlich sorption coefficient with the units of (mL/g)^n^ and *n* is the Freundlich exponent (dimensionless) [26]. The Freundlich isotherm assumes that adsorption takes place on heterogeneous surfaces [39]. The Freundlich isotherm has been shown to demonstrate that the ratio of solute adsorbed onto a given mass of the adsorbent to the amount of concentration of the solute in solution is not a constant at different solution concentrations [40]. 

If *n* = 1, then the Freundlich isotherm becomes a linear one and this indicates partitioning as the sorption mechanism (see Section 2.3 for details). In this case, the adsorption sites have equal adsorption energies. This is a common phenomenon at very low solute concentrations and low sorbent loading. When the sorption energy is directly proportional to the surface concentration of the sorbate, then there is an increase in the marginal sorption energy which in turn causes an increase in the surface sorbate concentration and (1/*n*) > 1. Under such circumstances, the Freundlich isotherm becomes concave, indicating the solvent affinity type of isotherm (S-Type). This was reported for example by Tandlich and Balaz [26] for the sorption of biphenyl onto kaolinite. The *K*_f_ value was equal to 9.30 ± 8.40 (mL/g)^(1/2.57)^, while the (1/*n*) value was equal to 2.57 [26]. This type of isotherm is observed is the rate constant of adsorption is higher than the desorption counterpart [26]. 

On the other hand, the Freundlich isotherm is of the L-type, if (1/*n*) is lower than 1 and it becomes convex in shape. This implies that with the increase of the surface concentration, the marginal sorption energy decreases [41,42]. Practically, this can take place either if the competition for the sorption binding sites between the sorbate and its solvent is minimized, or if the sorbate molecules are planar in shape. Such observations were made by Bonin and Simpson [63]. These authors studied the sorption of 17*β*-estradiol, estrone, and 17*α*-ethinylestradiol onto sand, kaolinite and montmorillonite and whether the process was best described by either Langmuir or Freundlich model. Sorption antagonism was observed between various sorbates also as a function of the soil organic carbon content and the montmorillonite content [63].

Polati *et al*. [64] conducted a detailed investigation into the sorption-desorption of 3-chloroaniline, 3,4-dichloroaniline, 2,4,6-trichloroaniline, 4-chlorophenol, 2,4-dichlorophenol and 2,4,6-trichlorophenol onto the following matrices: KGa-1 kaolinite and sodium-exchanged montmorillonite SWy-l [64]. The initial dissolved sorbate concentrations ranged from 10.0 mg/L to 200.0 mg/L. There were no significant differences between the sorption capacities of the two clay sorbents [64]. The highest sorption capacity of 8 mg/g clay mineral was recorded for the most hydrophobic compound, namely 2,4,6-trichloroaniline [64]. Sorption of all studied compounds onto kaolinite was irreversible and the sorption isotherms followed the Langmuir and Freundlich models equally well. Given this observation, kaolinite could be used as an effective matrix in remediation operations for aromatic hydrocarbons and chlorinated derivatives. 

Chu *et al*. [65] studied the effects of vegetative management, soil properties and high-molecular weight dissolved organic matter on the soil sorption of sulfamethazine. Three soils were sampled and tested within a dissolved concentration interval of 2.5–50 μmol/L of the antibiotic. The Freundlich isotherm model best described the sorption of sulfamethazine with *K*_f_ values ranging from 2.754 to 8.511 (mL/g)^0.59–0.79^ and the Freundlich exponent varying between 0.59 and 0.79 [65]. The apparent soil/water partition coefficients indicated that vegetative management, the concentration of organic carbon, soil pH and the initial sulfamethazine concentration were the most important factors governing the soil sorption of the antibiotic [65]. In clay soils, the sulfamethazine sorption was virtually independent of the soil pH, and the same observation was made about the antibiotic sorption in all three soils with respect to dissolved organic matter [65].

In the concentration range from 1 μg/L to 9 μg/L, cypermethrin (a pesticide) followed the Freundlich isotherm and its affinity to mineral surfaces decreased in the following order: corundum > quartz > kaolinite > montmorillonite > goethite [66]. This pesticide can form diastereoisomers which follow linear sorption isotherms on quartz, corundum and goethite [66]. The isotherms became non-linear for the *cis* A and *trans* C isomers on kaolinite and montmorillonite. Kaolinite proved to have the highest sorption affinity towards *cis* B and *trans* D isomers [66]. This supports the above conclusion about the possibility of applying kaolinite in remedial operations for the removal of aromatic hydrocarbons, their chlorinated derivatives and pesticides. 

### 2.3. The Linear Isotherm

In linear isotherms, sorption of HOCs onto soils and clay minerals occurs due to partitioning and is described by the soil/water sorption partition coefficient for the respective sorbate, *i.e.*, *K*_d _(units mL/g or mmol/g) [67,68]. Mathematically, *K*_d _can be defined using Equation (4) as shown below:

*q*_e_ = *K*_d_ × *C*_e_ (4)

All terms in Equation (4) have the same meaning as in Section 2.1 and Section 2.2 and the unit of the linear sorption partition coefficient *K*_d_ is mL/g or L/kg. The linear sorption partition coefficient is the slope of the linear sorption isotherm [26].

Linear sorption isotherms have been reported by Tandlich and Balaz for biphenyl on the illite-rich soil from North Dakota and a commercial sodium bentonite sample [26]. On the illite-rich soil, the *K*_d _value for biphenyl was equal to 42.7 ± 1.8 mL/g after 6 days and this value increased to 120 ± 8 mL/g after 21 days of the sorbate/sorbent contact time [26]. With the commercial sodium bentonite (montmorillonite), the *K*_d_ value was virtually independent of the contact time, as the respective *K*_d _value was equal to 20.3 ± 0.3 mL/g after 6 days and to 23.0 ± 1.1 mL/g after 21 days of sorbent-sorbate contact [26]. After normalisation to the solid/liquid ratios for both sorbents, the six-day contact time, most likely resulted in the sorption onto the internal surface of the clay mineral crystal lattice, while the 21 day period indicated when the soil organic carbon become the dominant sorption site for aromatic HOCs [26]. For other HOCs besides biphenyl, such sorption behaviour could be described a combination of linear and Freundlich isotherms can be observed at low organic carbon concentrations [69]. Mathematical of the resulting isotherm is similar to Equation (2). 

Wang *et al*. [70] found that nonylphenol, if sorbed onto the surface of kaolinite at concentrations of 1.0 mg/L, inhibited successive sorption of phenanthrene which is a polyaromatic hydrocarbon. If the dissolved concentration of nonylphenol in the aqueous phase reached 10 mg/L and this solution was in contact with kaolinite prior to phenanthrene sorption, then the apparent *K*_d_ values for phenanthrene increased in comparison to the 1.0 mg/L conditioning [70]. The most likely explanation for this observation is that at the initial nonylphenol concentration of 1.0 mg/L, the surfactant and phenanthrene were sorption antagonists. On the other hand, if the initial nonylphenol concentration increased to 10 mg/L, then enough surfactant was likely sorbed onto the kaolinite surface to form nonylphenol hemimicelles or micelles there. If this was the case, then micellial nonylphenol structures would have formed a hydrophobic phase at the kaolinite surface, *i.e.*, thus facilitating increased sorption partitioning of the phenanthrene molecules onto the kaolinite surface. The presence of microbial cells on the surface of the soil mineral phase has been shown to influence phenanthrene sorption [71]. Thus, the combination of the kaolinite with surfactants could provide a viable remediation technology for HOC elimination from the environment. 

## 3. HOC Binding to Soil Components and Sediments

### 3.1. HOC Binding to Soil Organic Matter/Carbon

Soils are mixtures containing soil micro- and macrobiota, organic components (humin, humic acids and fulvic acids) and minerals [21]. Sorption of HOCs onto soils is dependent on the amount and type of the soil organic matter (SOM) in a given sorbent [47,67], the nature and composition of the soil mineral phase, the water/moisture content of particular soil(s) and the liquid medium that the soil in question is in contact with and that the HOCs are sorbing from onto the given soil [35]. The structure of a SOM will depend on the geographical location of the soil biotope, geological history, bedrock material and the origin of the soil in question [67]. All these factors will have a significant influence on the HOC sorption onto the studied soils [67]. The main compounds and polymers contained in the SOM including the following chemicals [27,43,72]: lignin, proteins, polysaccharides, black carbon, humic acid, humin and fulvic acids. These fractions form the SOM and each of them is a potential sorbent for HOCs and organic chemical compounds/pollutants in general [73].

Particulate organic matter (POM) is part of the SOM and it contains among other things organic debris with particle diameters up to 0.05 mm [43]. POM has been shown to play an important role in the HOC sorption to soils [43]. As with other SOM components, the HOC sorption onto POM will be related to the sorbent’s chemical composition which in turn is a function of the soil’s origin and humification history [43]. As a result of this, sorption of HOCs on POM will differ between individual soils and the type of debris of organic origin found in particular soils [43]. Guo *et al*. [43] studied the sorption of naphthalene, phenanthrene, and pyrene onto POM. They compared the sorption of these polycyclic aromatic hydrocarbons onto four soils from different origins to illustrate the effect of the different compositions of POM of these soils.

The Freundlich isotherm (Equation (3)) was used to analyse the data and the intensity of the HOC sorption was evaluated based on the *n* values derived from experimental data using non-linear regression [43]. This is based on the fact that the *n* values describe the site energy distribution on the soil surface [43]. Therefore the smaller the *n* value, the more heterogeneous the sorption site energy distribution. In the experiments of Guo *et al*. [43], the POM content did not vary significantly in the four soils examined, with *n* varying from 0.86 to 1.00 [43]. POM 1 contained mobile aliphatic carbon atoms and alkyl groups and the respective *n* values were closer to 1.0, *i.e.*, the sorption onto this type of POM was closest to the partitioning mechanism than was the case for POMs 2–4 [43]. Freundlich exponent values ranged from 0.83 for naphthalene to 0.97 for phenanthrene, while the respective value for pyrene was equal to 1.00 [43]. These results indicate that the number of aromatic rings in a polyaromatic hydrocarbon and the molecular volume likely influence the sorption of polyaromatic hydrocarbons onto soils.

Sorption of cypermethrin to POM was also influenced by the nature and origin of POM; and the extent increased with the increasing coating of the clay mineral montmorillonite by the humic material [74]. The affinity of the HOC is influenced by the composition of the NOM, the NOM-chemical contact time and the solute properties [75]. Comparison of the significance of clay minerals and SOM are indicated by studies such as that by Tandlich and Balaz [26]. The authors found that the soil organic carbon became the dominant sorption sites for biphenyl after 21 days of sorption. Up to 6 days of the contaminant soil contact, the external surfaces of the kaolinite provided a temporary sorption site for biphenyl, with the internal surfaces of illite and bentonite becoming more important between 6 and 21 days [26]. Soil organic carbon contains various sub-components with varying affinity for HOCs’ sorption [76]. One such component is soot/black carbon [77]. The HOC soil sorption is affected by the total concentration of SOM, if the concentrations of soil organic carbon (SOC) exceeds 0.01%–0.2% [27,78,79]. Differences in sorption are based on factors such as hydration which is described more in the next Section(s). 

### 3.2. Effect of Hydration of the Sorbent and Medium of Sorption.

Hydration can affect the SOM structure and thus the HOC sorption and any sorption antagonism of any HOC mixtures binding to the SOM. Graber *et al*. [80] studied the effect of Pahokee peat hydration on sorption of phenol, pyridine and atrazine to this mineral-free SOM. The sorbates were dissolved in water, *n*-hexadecane and *n*-hexane [80]. Sorption from aqueous solutions followed the linear sorption isotherms, while Langmuir sorption isotherms were observed in the hydrocarbon media for all three pollutants [80]. For the Langmuir isotherms, the respective *q*_m_ values were determined to be equal to 1397 ± 137 mmol/kg for phenol, 1082 ± 131 mmol/kg for pyridine and 448 ± 55 mmol/kg for atrazine [80]. The molecular volume of atrazine is higher than the corresponding values for phenol and pyridine, and it was quoted as the reason for the lowest *q*_m _value out of the three pollutants studied [80]. This is likely caused by the fact that the majority of the atrazine sorption takes place on the outside of the peat particles [80], in contrast to the other two pollutants where the molecular volume likely allowed for pollutant diffusion into the inside of the Pahokee peat particles [80]. 

*N-*hexadecane and *n*-hexane are inert and saturated hydrocarbons and cannot participate in non-covalent interactions such as hydrogen bonding or as Lewis acids and bases in reactions [68]. If sorption from these solvents onto soil particles takes place, non-covalent interactions responsible for sorption will be predominantly determined by the covalent structure of the sorbate(s) and the molecular composition of the sorbent. The type of interactions that are responsible for the sorption of a given HOC onto NOM can thus be deduced by studying its sorption onto the (hydrated) sorbent from an inert solvent. As hydration can stimulate the occurrence of non-covalent interactions such as hydrogen bonds, their significance in sorption can be deduced from sorption of HOCs with varying structures onto Pahokee peat from *n*-hexadecane [68,71]. Use of this solvent eliminates the influence of polarizability of the tested HOCs on their sorption onto Pahokee peat [68,71]. Under the same conditions, the effect of the compound’s structure on its sorption onto the NOM can be elucidated, along with the structural features in the NOM structure being characterised [68,71].

Using the study of Graber *et al*. [80], the abovementioned concept was applied to phenol (a hydrogen-donating compound in hydrogen bonding) and pyridine which is a hydrogen acceptor in hydrogen bonds [80]. Graber *et al*. [80] concluded for the sorption data that the sorption of HOCs from the water system was much higher than the sorption from the hydrocarbon system due to an increased number of sorption sites in the Pahokee peat upon hydration [80]. The hydrated peat structure contains more water molecules than the air-dried matrix. This leads to more hydrogen-bonding potential and thus provides for the increased sorbate mass transfer through/into the Pahokee peat structure based on hydrogen bonds [80]. Such hydration is facilitated by the presence of hydrogen-bonding groups, such as COOH in the structure of the peat. This in turn increases the number of available sorption sites upon hydration of Pahokee peat more, than upon its solvation with organic solvent. More sorption sites lead to higher sorption capacities, *i.e.*, likely resulting in the linear sorption isotherms under conditions of hydration of Pahokee peat in aqueous environments [80]. Similar solvation does not take place in the *n*-hexadecane, thus lowering sorption uptake for phenol and pyridine [80]. 

These conclusions about the role of hydration in the sorption of HOCs onto the soil organic carbon/matter are supported by the findings of Borisover *et al*. [73]. They examined the sorption of *m*-nitrophenol from *n-*hexadecane, *m*-nitrophenol from hexane, nitrobenzene from hexadecane and acetophenone from *n*-hexadecane, benzyl alcohol from *n*-hexadecane onto NOM [73]. Sorption from water was also studied for all solutes [73]. *N*-Hexadecane and *n*-hexane are hydrophobic and were considered the dry inert systems [73]. It was found that the sorption of organic compounds from hydrocarbon solutions on dried NOM was much slower as compared to sorption of organic compounds by the NOM sorbent from water. This is demonstrated by the observation that the apparent sorption equilibrium for *m*-nitrophenol was reached at about 50 h from the aqueous phase as compared to between 300 and 600 hours in *n*-hexane and 700 hours in *n*-hexadecane [73]. They attributed this to the poor solvation of the sorbent in the hydrocarbon phase resulting in the “rigidity” of the sorbent, *i.e.*, formation of new sorption sites for hydrogen-bonding compounds in the hydrated Pahokee peat as compared to peat exposed to the dry peat matrix [73]. 

Borisover *et al*. [81] examined twelve systems with the following combination of sorbate/sorbent/ solvent: *m-*nitrophenol/humic acid/water, *m*-nitrophenol/humic acid/*n*-hexadecane, *m*-nitrophenol/ humin/water, *m*-nitrophenol/humin/*n*-hexadecane, nitrobenzene/humic acid/water, nitrobenzene/ humic acid/*n*-hexadecane, nitrobenzene/humin/water, nitrobenzene/humin/*n*-hexadecane, acetophenone/humin/water, acetophenone/humin/*n-*hexadecane, benzyl alcohol/humin/water, and benzyl alcohol/humin/*n*-hexadecane. From their study, the authors concluded that hydration of the NOM may cause up to 2–3 orders of higher sorption of organic compounds in comparison to wetting by hydrocarbon solvents [81]. Acetophenone and benzyl alcohol sorption was higher in the aqueous conditions as compared to *n-*hexadecane [81]. Lower polarity of the HOCs resulted in stronger sorption to dry humin, as demonstrated by the strength of sorption to dry humin decreased in the following order [81]: nitrobenzene > *m*-nitrophenol > acetophenone > benzyl alcohol. Sorption of compounds with strong specific interactions, such as H-bonding (benzyl alcohol, *m-*nitrophenol), is significantly influenced by hydration [81]. For acetophenone and nitrobenzene there was a decrease in sorption upon hydration, whereas the opposite was recorded for benzyl alcohol and *m*-nitrophenol [73,81]. Thus, hydration of NOM or SOM leads to formation of new sorption sites, but access to them is controlled by the sorbate’s molecular volume and its functional groups. 

### 3.3. Effects of Temperature, Ionic Strength and pH on the HOCs’ Sorption

In a study to determine the effects of temperature on sorption by Zhang *et al*. [60], the Freundlich isotherm best described the sorption data of naphthalene and phenanthrene onto soils between 15 to 35 °C [60]. It was noted that *n* was directly proportional to the incubation temperature as it increased from 0.713 to 0.893 for naphthalene and from 0.550 to 0.756 for phenanthrene, respectively [60]. *K*_f_ for naphthalene decreased from 106.8 to 42.8 in the respective units, while the *K*_f_ value for phenanthrene decreased from and 932.7 to 568.9 (mL/g)^0.55–0.893^ [60]. According to Equation (3), *K_f_* and *n* are isotherm constants for a given sorbate and sorbent; and they indicate the capacity and intensity of the sorption at a given and constant temperature. Zhang *et al*. [60] also reported that as temperature increases *n* increases, *i.e.*, the solute-solvent interaction strength increases which in turn leads to a decrease in *K_f_*. Similarly, an inverse correlation between the temperature and the sorption partition coefficient normalized to the soil organic carbon content (*K_oc_*) was reported [60].*_._* The most common explanation is the increase I n the solute’s relative affinity for liquid phase in comparison of the solid phase. The effect of pH has been studied mainly in the soil/aqueous systems. The effects pH on the sorption of 4-phthalic acid ester, dimethyl phthalate (DMP), diethyl phthalate (DEP), diallyl phthalate (DAP) and di-*n*-butyl phthalate (DBP), on three soils have been studied [82]. The aqueous phase pH was set to 4.0, 5.5, 7.0, 8.5 and 10.0. An increase in pH leads to the decrease in the *K_f_* as shown in Table 1. At pH 4.0, the maximum *K*_f_ values were observed, and thus the sorption capacity of the soils for phthalic esters, was indirectly proportional to the aqueous phase pH [82]. This was attributed to the increase in the dissociation/ionisation of the functional groups on SOM which leads to the increase in the charge in the SOM structure [82]. This resulted in the decreased sorption of phthalic esters to the soils studied as these are non-ionised HOCs. Similar observations were reported in other studies [46,83,84].

**Table 1:** The *K_f_* values of the phthalic acid ester in relationship to pH (adapted from reference [82].

Compound	DMP *K_f_*	DEP *K_f_*	DAP *K_f_*	DBP *K_f_*
pH 4.0	5.31	9.87	33.1	161
pH 5.5	4.64	8.59	28.3	147
pH 7.0	3.18	6.38	21.7	128
pH 8.5	2.70	5.85	19.6	97.2
pH 10.0	2.54	5.85	18.3	91.3

Salting out effect, *i.e.*, the increase in the apparent sorption of non-ionic HOCs into kaolinite in the case increased aqueous phase ionic strength was observed for endrin by Peng *et al*. [85]. The sorption isotherm was linear and troughs were observed at pH = 5.4 [85]. Hydrophobic and ion-dipole interactions were found to the main non-covalent interactions involved in the endrin sorption to kaolinite [85]. Finocchiaro *et al*. [86] examined the relationship between the following soil properties: pH, the organic carbon concentration, the content of amorphous iron oxides and the content of clays, and the soil sorption uptake of molinate, terbuthylazine, bensulfuron methyl and cinosulfuron. Extent of sorption of molinate and terbuthylazine was directly proportional to the concentration of organic carbon and the content of amorphous iron oxides [87]. On the other hand, sorption of bensulfuron-methyl to be a function of the soil pH, the organic carbon concentration and the clay content [86]. Finally, the cinosulfuron sorption was positively correlated with the soil pH [86]. Thus all these variables together with the covalent structure of the HOCs must be taken into account when conducting a sorption experiment

Behra *et al*. [87] showed that tributyltin sorbed onto mineral surfaces via cation exchange of the monovalent cation of the tin complex for H^+^ and Na^+^, if the pH of the aqueous phase was equal to 6.0 or less. Sorption capacities of mineral surface for cationic version of tributyltin decreased from pure quartz > treated sand > natural sand >> kaolinite [87]. The XPS results indicated that tributyltin sorbs first by creating a monolayer on the mineral surface which thus becomes hydrophobic if the sorbate concentrations reach 100 μM or more [87]. Further sorption past this point occur via dissolution of the tributyltin molecules in the hydrophobic phase formed by the butyl side-chains of the organometallic species, *i.e.*, the mechanism of sorption is likely surface condensation [87].

### 3.4. Interaction of HOC with Soil Mineral Phase

#### 3.4.1. Major Soil Mineral Components

The mineral particulate nature of soils is based on diameter and consists of sand (50 µm–2 mm), silt (2 µm–50 µm) and clay, with diameters below 2 µm [42]. The dominant mineral structures in clay soils are silicates and aluminosilicate mineral structures [88]. Butachlor (CAS number: 23184-66-9) belongs to the acetanilide class of herbicides and its sorption onto montmorillonite, kaolinite, the calcium montmorillonite, calcium kaolinite amorphous hydrated aluminium and iron oxides have been reported [89]. The most important clay mineral with respect to butachlor sorption was montmorillonite [89]. This indicates the important role of expanding minerals in butachlor sorption onto soil particles. Structure of the sorbate and the concentration of the soil organic carbon have a strong influence on the sorption of the HOCs onto clay minerals as demonstrated for trichloroethylene [90]. The sorption isotherm can be linear, as shown for trichloroethylene [15,90], or non-linear as reported in selected cases for perchloroethylene [15]. The details of the non-covalent interactions between organic sorbates and clay minerals with specific focus on kaolinite are described below.

#### 3.4.2. Hydrophobicity of Siloxane Groups in Clay Soils in the Sorption of HOC

Siloxane groups are composed of alternating silicon and oxygen atoms connected by a covalent bond [-Si-O-Si-] [42,91]. As summarised by Tandlich and Balaz [26], it has been shown by some studies that siloxane surfaces inside the crystal lattice of expandable clays can be without net charge [92]. Theoretical calculations also suggest that the O atoms that are located in the interlayer spaces in the clay crystal lattices are hydrophobic in nature [52]. These findings provide possible sites for sorption of aromatic hydrocarbons [93]. On the other hand, siloxane groups in clay soils can also be hydrophobic in nature, if they do not contain isomorphous substitution [42,91]. In the study done by Su *et al.* [91] on the adsorption of poly(ethylene oxide) on smectite, they concluded that an interaction between the siloxane groups of the smectites with the -CH_2_-CH_2_- of the poly(ethylene oxide) occurred while the poly(ethyleneoxide) hydrophilic ether group formed a hydrogen bond with the OH structures on the smectite. This showed that the hydrophobic part of poly(ethyleneoxide) had a higher affinity for the siloxane groups in the smectite soils [91].

Jaynes and Boyd [93] studied the nature of siloxane in the modified smectite soils by converting them to organo-soils by replacing the hydrophilic, inorganic exchange cations of a series of smectites with the small, hydrophobic organic cation, trimethylphenyl ammonium (TMPA). This limited the adsorption sites to the TMPA cations and the siloxane oxygen surfaces. Sorption of benzene, toluene, ethylbenzene, propylbenzene butylbenzene and naphthalene were studied on Wyoming montmorillonite (SAC), an Arizona montmorillonite (SAz) and Washington nontronite (SWa) [93]. The results of their experiments showed that aromatic hydrocarbons can effectively adsorb the siloxane surfaces of the smectites if hydrophilic, inorganic exchange cations are replaced with small, hydrophobic organic cations [93]. The Langmuir isotherm parameters for benzene and propylbenzene obtained suggested that adsorption occurred on the clay surface, and not on the organic phase derived from the TMPA cations. It was discovered that adsorption was inversely proportional to the TMPA from the sorption isotherms. This was because as TMPA content decreased, sorption increased as layer charge increased. They concluded that the organic compounds adsorbed to the siloxane surfaces and this demonstrated the hydrophobicity of the siloxane surfaces in smectites [26,93].

For organic cations, the results of flow-through analysis indicate that sorption isotherms are linear at sorbate concentrations lower than 10% of the cation-exchange capacity for illite and kaolinite, and up to just below 1% of the cation exchange capacity for bentonite [94]. A significant influence of the liquid phase used to leach the solid/clay matrix on the cation sorption by clay matrices was observed [94]. The extent of this effect required the use of correction factors which were based on empirical measurements [94]. The variability in sorption affinities among kaolinite, illite and bentonite could be eliminated by normalisation of the cation sorption to the cation exchange capacity of the given clay mineral [94]. After such normalisation, the importance of organic matter and soil minerals in organic cation sorption was comparable, with the exception of quaternary ammonium salts [91]. This observation stresses the potential significance of clay minerals in pollutant retention as indicated by the results of Tandlich and Balaz [26]. 

TMPA is a small organic cation. It forms monolayers between the inter layers of the smectite soil. This occurs in both high charge and low charge smectites. The TMPA cations are physically more isolated in low charge smectites than in high charge smectites, hence this leads to more of the inter layer clays surface being available for adsorption. The interlayer of the smectite is composed of the siloxane layers hence the adsorption was attributed to the siloxane bonds. The reduced-charge montmorillonites were prepared from the SAz using Li-saturated and Na-saturated clay suspensions in the ratios 0.3 Li/0.7 Na, 0.6 Li/0.4 Na, 0.8 Li/0.2 Na, and 1.0 Li/0.0 Na. The soils were respectively coded as 0.3 Li-250, 0.6 Li-250, 0.8 Li-250, and 1.0 Li-250. From the adsorption isotherms of benzene, toluene, ethylbenzene, propylbenzene, butylbenzene, and naphthalene onto SAz-TMPA, 0.3Li-250 SAz-TMPA and 0.6Li-250 SAz-TMPA, it was concluded that the more reduced the SAz the higher the sorption of the organic compound [93]. 

## 4. Kaolinite

### 4.1. Structure of Kaolinite

Kaolinite is a naturally occurring inorganic polymer. It consists of siloxane and gibbsite-like layers. Its chemical formula is Al_2_[Si_2_O_5_](OH)_4_. It consists of a 1:1 octahedral alumosilicate sheet with aluminium cations bonded to another tetrahedral sheet with silicon cations [95,96]. These sheets are stacked on top of each other and the adjoining layers form van der Waals forces and hydrogen bonds because of the availability of OH groups and oxygen atoms in the two adjacent layers [95]. The hydroxyl functional groups on kaolinite are the most reactive [95,97]. They often take part in various chemical reactions including ion exchange [94,97]. The inability of kaolinite to expand due to hydrogen bonds [26] means that its internal surface area is negligible as compared to the total specific surface area [93], leading to the conclusion that sorption of HOC takes place mostly on the outer surface of kaolinite [26,98].

The crystal lattice of kaolinite is neutral as compared to other clay soils, and its two well-defined layers, alumina surface and silica surfaces provide two different potential surfaces of adsorption because of the hydroxyl groups and the silica-oxygen bridged surfaces [99]. Kaolinite has a high affinity for organic compounds as compared to other clay surfaces especially illite [100,101]. According to van Duin *et al.* [99] this could be due to the neutrality of the kaolinite lattice and due to the close proximity of charged counter-balancing ions to the illite surfaces rendering it a low affinity for less polar organic compounds. Saada *et al*. [100] focused on the hydrophilicity and hydrophobicity of illites and kaolinite. They concluded that only 25% of the kaolinite surface is hydrophilic and the remaining part is either neutral or hydrophobic compared to illites which are 40% hydrophilic [100]. They concluded that as the hydrophilicity of asphaltene increases, the adsorption capacities decreases. Wettability by oils was found to be high for kaolinite surface [100].

The infrared bands of kaolinite between 3700 and 3620 cm^−1^ correspond to the well-crystallized structure of kaolinite [101]. Cheng *et al.* [102] contradicted Suraj *et al*. [103] where the bands at 937 and 914 cm^−1 ^were attributed to OH bending vibrations and bands at 983 and 1035 cm^−1 ^were attributed to the Si-O-Si in-plane vibrations [102]. As described above, the Si-O-Si represents the siloxane bonds which are hydrophobic and will have a bearing on the sorption of HOC. Table 2 below shows the spectral wave lengths of kaolinite as studied by Suraj *et al*. [103].

**Table 2:** Band assignments for kaolinite soil [103].

Wave Number (cm^−1^)	Assignments
3700	Inner surface -OH stretching vibration
3620	Inner -OH stretching vibration
1114, 1035, 1010	Si-O bending vibrations
938, 918	AI-OH bending vibration
792, 754	Si-O-Al compounded vibrations
692	Si-0 stretching vibration

### 4.2. Interaction of Kaolinite with Organic Molecules

In a limited number of cases, HOCs can interact with kaolinite through intercalation between the kaolinite layers [104]. Examples include the hydrogen-bonding compounds which undergo interactions between hydroxyl groups of a octahedral alumosilicate sheet and the tetrahedral sheet with silicon, *i.e.*, N-methylformamide (NMF) and dimethylsulfoxide (DMSO). The other reason is that the internal surface area of kaolinite is very small [81], hence only smaller molecules can intercalate. Specific surface area of the sorbent is also a factor to consider. Different methods of measuring specific surface area have been adopted. The ethylene glycol monoethyl ether (EGME) method has been used to calculate both the external and internal specific surface area of the soils (interlayer surfaces of soils and clays) [26,105,106]. In different studies done using kaolinite from different locations it was found that the specific surface area of kaolinite was 25.5 m^2^·g^−1 ^[107], 5.9 m^2^·g^−1 ^[108] and 15 m^2^·g^−1 ^[106]. It has been stated in the literature that non-expanding soils like kaolinite have SSA values ranging from 10 m^2^·g^−1^to 40 m^2^·g^−1 ^[106].

Intercalation of NMF or DMSO is an intermediate state in the intercalation of other guest species, [109]. The process breaks down the hydrogen bonds linking the gibbsite and the siloxane layers of kaolinite [102], making kaolinite a single layered mineral [95,110,111]. New hydrogen bonds are formed between the inserted molecules and the crystal lattice of the clay mineral [104] and the intercalation process has three stages [112]. The intercalation of kaolinite by organic compounds has been studied in recent years [99]. In the study done by Komori *et al*. [109] on the intercalation of alkyl amines (C*n*N; *n* is the carbon number in alkyl chain) in kaolinite they found out that octylamine was bound to the Al_2_[Si_2_O_5_](OH)_4 _in the ratio of 2.4 moles of octylamine to 1 mole of kaolinite. Intercalation occurred via an intermediate step where kaolinite/methanol was made from kaolinite/NMF and used as the intermediate. Intercalation was done up to alkyl C_18_N. These intercalation compounds are applicable as other effective intermediates and extend the variation of kaolinite/organic intercalation compounds. Secondary and tertiary amines could not be intercalated in kaolinite because they lack the ability of forming hydrogen bonds with the hydroxyl groups and basal O atoms of kaolinite [109].

Intercalation of alkylamines was also attributed to the Van-der-Waals interactions between alkyl chains. This was concluded because of the quick deintercalation of C_6_N when exposed to air [108]. Erten *et al*. [113] showed that residual amounts of NAPL that can’t be removed from clay media by consolidation settlement are on the order of 0.1 g NAPL/1 g soil particulate matter. Clay minerals can be ion-exchanged with long-alkyl cations which leads to the formation of so-called “organophilic clays” [31]. Such matrices have been shown to adsorb up to 0.93 g NAPL/1 g of organophilic clay [114]. This has been reported to have a significant effect on the perturbation of the NAPLs from Soltrol 130 through the kaolinite matrix [114]. Organophilic clays, such as kaolinite modified with cetyltrimethylammonium bromide have been shown to exhibit linear sorption uptake for hydrophobic chemicals, as demonstrated for chlorobenzenes [115]. The apparent soil/water distribution coefficient has been shown to increase from kaolinite to bentonite (montmorillonite), *i.e.*, the surfactants coat both internal and external surface and provide additional surfaces for chlorobenzene dissolution [115]. 

For ionic liquids, Mrozik *et al*. [116] found that sorption on kaolinite surfaces took place *via* multi-layer formation, analogous to the tributyltin results of Behra *et al*. [87]. The average free energy of sorption values indicated that the sorption mechanism was overlapping of electrostatic interactions between the sorbate and sorbent; and physical sorption [116]. The chemistry of the kaolinite surface has been shown to determine the extent of dye sorption onto the clay mineral. This can be demonstrated by the increase in the sorption capacity of the kaolinite from the Delta State, Nigeria, for aniline blue from 1,666 to 2,000 mg/kg upon surface modification with sodium tetraborate [117].

## 5. Experimental Determination of Sorption Co-Efficient and Sorption Kinetics

### 5.1. Experimental Determination of Sorption Coefficient

Batch equilibration (BE) is a common experimental method of determination of sorption coefficients. Important factors to consider in the batch equilibration method are: (i) equilibration time, (ii) sorbent and solute concentration, and (iii) temperature [102]. For reliable results these factors have to be determined for the solute and the sorbent in question. After this *K_d_* can determined in the method outlined below. A soil/sediment is weighed and placed in a vial or container. A known concentration of the solute, normally dissolved in the solvent in question, is added. The vial is not filled to capacity hence leaving room for the volatilization of the solute into the headspace of the vial. The vial is sealed and shaken until equilibration is reached. The vial is then centrifuged and the two phases are separated and examined for the concentration of the solute. The amount of the solute which is sorbed onto the soil/sediment may also be calculated by finding the difference between the initial concentration of the solute and the final concentration of the solute. The *K_f_* and *K_d_* are calculated by manipulating the Freundlich and Langmuir isotherms, respectively [26,35]. 

The sources of error in the batch equilibration method are the length of the experiment *i.e.*, time to reach equilibration and failure of the complete separation between sorbent and the phase in which the sorbent is dissolved [35]. For experiments where a longer time is required to reach equilibration, measures should be taken to avoid physical losses of the solute for example through volatalisation [26,35]. To avoid degradation of the solute by soil microorganisms autoclaving has been used [118]. This is important to avoid loses of solute through degradation. Sodium azide has also been shown to render effective sterilization without altering the chemical structure of the soil [37]. Soil: solution ratio is also paramount in this method. This ratio can be altered to effect a significant change in the difference between the initial concentration of the solute and the final concentration of the solute hence more reliable results. In the batch method it is important to adjust this ratio to between 20% and 80% [102] and in some cases 15%–70% [39] of the solute is removed to minimize errors. However it is important to choose this ratio carefully because of the solids’ effect due to the presence of non-settling particles. 

### 5.2. Sorption Kinetics

For sorption measurements equilibrium should be reached [42]. This is because there is a saturation of sorption sites; hence no further sorption is expected to occur. This is where a “steady state” concentration has been established. Equilibrium is reached at different times depending on the characteristics of the sorbent and sorbate. as well as the characteristics of the media from which the solute is dissolved. Equilibrium times range from 1 h to a few hours to days, months and years, depending on the structure of the compound and media/sorbent where the individual equilibration takes place [18,37,38,40]. After apparent equilibrium has been reached the amount of solute removed by the sorbent is calculated. 

### 5.3. Relationship between Hydrophobicity of the Solute and Sorption

When little or no empirical data are available quantitative structure–activity relationships (QSARs) are reliable tools for the hazard assessment of organic chemicals [2,119]. Organic compounds are characterized as more or less lipophilic. Hydrophobicity is important in the QSAR and therefore it is important to determine the hydrophobicity of an organic compound [120]. Lipophilicity/hydrophobicity can be measured using the thermodynamic distribution ratio of the solute between two immiscible solvents. The distribution ratio is defined as the ratio of equilibrium concentrations of a substance distributed in any binary system consisting of two largely immiscible solvents [121]. The distribution ratio that can be called the partition co-efficient *P,* which is defined as the ratio of the equilibrium concentration of a chemical in the two adjacent phases, 1-octanol and water [122]. *P* is a routine measurement of a compound’s hydrophobicity [123]*.*

It is possible to investigate the effects of different compound characteristics such as presence of aliphatic moieties, aromatic moieties, hydrogen bonding potential and 1-octanol-water distribution (log*K_ow_*) on sorption [69]. The partition in water and organic solvent systems was reported by Colander in 1947. He reported that the relationship between partition coefficients of various organic solvents in a two phase system of water/organic solvent, and the partition coefficients of the same solutes in a different organic solvent/water system were linear using the logarithmic scale; he thus derived the Colander equation [124,125,126]. Sorption of organic compounds can be regarded as the partitioning of the hydrophobic chemical between and aqueous and organic phases (hydrophobicity) [35]. Hydrophobicity is often quantified using the log*K_ow_* term which stands for the decadic logarithm of the 1-octanol/water partition coefficients. Octanol is a model of the hydrophobic phase and the organic matter found in soils and sediments may be equated to that of an organic phase in solvent extraction [35]. 

The sorption capabilities of kaolinite and montmorillonite to sorb HOCs with different lipophilicities is directly proportional to the lipophilicilty. As the lipophilicity increases, the amount of sorbed HOCs increases. Gianotti *et al*. [98] studied the sequestration of the organic pollutants 2,4,6-trichloroaniline (2,4,6-TCA) with 1-octanol/water partition coefficients (log*K_ow_*) of 3.74 and 4-chlorophenol (4-CP) with a log*K_ow_* of 2.49, using kaolinite and montmorillonite. The amount of 2,4,6-trichloroaniline retained by both soils was higher than that of 4-chlorophenol. It was concluded that the lipophilicity of 2.4.6 TCA (log*K_ow_* = 3.74) resulted in its greater affinity for the soils as compared to 4-CP (log*K_ow_* = 2.49) [98]. These results were similar to a reported study by Angio *et al*. [44] on the sorption of, 3-chloroaniline, 3,4-dichloroaniline and 2,4,6-trichloroaniline. A similar conclusion was reported for the sorption of aniline, atrazine, simazine, diuron and aromatic sulfonates as a function of hydrophilicity [40].

Sanchez-Martin *et al*. [127] studied the relationship between hydrophobicity (log*K_ow_*) of the pesticides and desorption of these pesticides from the different clay minerals modified with a cationic surfactant octadecyltrimetyl ammonium bromide (ODTMA). The pesticides used with their *a*re shown in the Table 3 below.

**Table 3:** The water solubility and log*K_ow_* of pesticides [127].

Pesticide	Water solubility (µg/mL)	log *K_ow_*
Penconazole	73	3.72
Linuron	81	3.00
Atrazine	30	2.50
Alachlor	240	2.63
Metalaxyl	8400	1.75

Desorption of these pesticides from natural unmodified soils followed a Freundlich isotherm with *R*^2^ ≥ 0.88, while the ODTMA modified soils had the same type of isotherm and *R*^2^ ≥ 0.92. The hysteresis coefficient, *H,* varied with the nature of the clay mineral and, mainly, with the hydrophobicity of the pesticide. Penconazole has the highest *H* because it’s the most hydrophobic pesticide and metal-axyl from ODTMA-montmorillonite. In the ODTMA-montmorillonite most of the pesticide was adsorbed in the interlayer space. There was no significant correlation between the organic matter content of soils or *K_ow_* value of pesticides and the adsorption of pesticides by clay minerals. Significant correlations between *K* values and organic matter were obtained with the ODTMA modified clays. *r^2^* values ranged between 0.81 and 0.96 for adsorption and the correlation between *K*_desorption_ and organic matter content was also high with r^2^ values ranging from 0.85 to 0.98. Correlations between *K, K*_desorption_ and *K_ow_* were related using Equation (5):

*K_des_* = 86.7 *OM* + 0.53 *K_ow_* − 1.57 (5)

Organic molecules which contain ionisable functional groups may also adsorb significantly onto mineral surfaces on the soils [41,128]. Studies have shown that the sorption of HOC is strongly dependent on organic carbon content. Combustion methods can be used to determine the organic carbon content (OC) [26]. In this study the relationship between *K_oc_* of naphthalene and aromaticity (AR) as compared with the predicted *K_oc_* from *K_ow_* [107]. Predicted *K_oc_* using was 1,130 mL/g. From the results above it is evident that *K_ow_*, the quality of the SOM and aromacity should be considered in order to accurately measure *K_oc_*. Sorption of naphthalene was directly propotional to aromaticity. Increase in aromaticity led to an increase in sorption and sorption increased with decrease in polarity of the SOM in the soil [121]. This was because naphthalene is a non-polar polyaromatic hydrocarbon. The relationship between *K_oc_* of naphthalene and effective polarity (PI) of the five soils is shown in equation 21 below [121], where PI is effective polarity.

## 6. Conclusions

Results of the review indicate that there is a possibility for the presence of hydrophobic siloxane groups on kaolinite and other clay minerals in soils. Thus soils can retain HOCsm, even if the concentration of the soils organic carbon is low. Data presented in this review provides some evidence about the wettability of clay minerals with nonpolar solvents and sorption of HOCs to the clay mineral can take place from aqueous and non-aqueous solvents alike. Therefore it is expected that clay minerals will sorb HOC such as NAPLs. Information presented in this review indicates that inorganic cations and surfactants sorbed onto the soils and clay minerals can alter the rate and extent of HOC sorption to these natural sorbents. In line with previous research papers and reviews, soil organic carbon plays a key role in the HOCs’ sorption to soils, but the extent will be strongly affected by the structure of the NOM, SOM, POM and the presence of soot. It is therefore imperative to characterise the soil organic carbon in a particular soil specimen. Due to the emerging pollutants such as mining extractants and antibiotics, more research is needed into predictive approaches such as QSAR in the context of the HOCs’ sorption onto clay minerals from aqueous and non-aqueous solutions.
